# Longitudinal experiences of Canadians receiving compassionate access to psilocybin-assisted psychotherapy

**DOI:** 10.1038/s41598-024-66817-0

**Published:** 2024-07-17

**Authors:** Sara de la Salle, Hannes Kettner, Julien Thibault Lévesque, Nicolas Garel, Shannon Dames, Ryan Patchett-Marble, Soham Rej, Sara Gloeckler, David Erritzoe, Robin Carhart-Harris, Kyle T. Greenway

**Affiliations:** 1 Department of Psychiatry, McGill University, Montréal, Canada; 2 Centre for Psychedelic Research, Department of Brain Sciences, Faculty of Medicine, Imperial College London, London, UK; 3 Lady Davis Institute at the Jewish General Hospital, Montréal, Canada; 4 Department of Psychiatry and Addictology, Faculty of Medicine, Université de Montréal, 2900 Boul. Edouard-Montpetit, Montréal, Canada; 5 Research Centre, Centre Hospitaller de L’Université de Montréal (CRCHUM), 900 Saint-Denis Street, Montréal, Canada; 6 Health and Human Services, Vancouver Island University, Nanaimo, Canada; 7 Northern Ontario School of Medicine University, Thunder Bay, Canada; 8 Marathon Family Health Team, Marathon, Canada; 9 Department of Neurology, University of California, San Francisco, USA; 10 School of Nursing and Midwifery, Queen′s University Belfast, Belfast, UK

**Keywords:** Psychedelics, Psilocybin-assisted psychotherapy, Distress, Palliative care, Quality of life, Psychology, Drug regulation

## Abstract

Recent clinical trials have found that the serotonergic psychedelic psilocybin effectively alleviates anxiodepressive symptoms in patients with life-threatening illnesses when given in a supportive environment. These outcomes prompted Canada to establish legal pathways for therapeutic access to psilocybin, coupled with psychological support. Despite over one-hundred Canadians receiving compassionate access since 2020, there has been little examination of these ‘real-world’ patients.

We conducted a prospective longitudinal survey which focused on Canadians who were granted Section 56 exemptions for legal psilocybin-assisted psychotherapy. Surveys assessing various symptom dimensions were conducted at baseline, two weeks following the session (endpoint), and optionally one day post-session. Participant characteristics were examined using descriptive statistics, and paired sample *t*-tests were used to quantify changes from baseline to the two-week post-treatment endpoint. Eight participants with Section 56 exemptions (four females, M_age_ = 52.3 years), all with cancer diagnoses, fully completed baseline and endpoint surveys. Significant improvements in anxiety and depression symptoms, pain, fear of COVID-19, quality of life, and spiritual well-being were observed. Attitudes towards death, medical assistance in dying, and desire for hastened death remained unchanged. While most participants found the psilocybin sessions highly meaningful, if challenging, one reported a substantial decrease in well-being due to the experience. These preliminary data are amongst the first to suggest that psilocybin-assisted psychotherapy can produce psychiatric benefits in real-world patients akin to those observed in clinical trials. Limited enrollment and individual reports of negative experiences indicate the need for formal real-world evaluation programs to surveil the ongoing expansion of legal access to psychedelics.

## Introduction

Psilocybin, a serotonergic psychedelic derived from ‘magic mushrooms’^1^, is classified as an illicit substance without recognized therapeutic value in most countries. In Canada, psilocybin and its active metabolite psilocin are currently classified as Schedule III substances, making them illegal to possess, obtain, or produce^2^. Despite its legal status, recent clinical trials have found that one or two doses of psilocybin can yield significant psychiatric benefits when paired with psychological support^3,4^.

These results have spurred investigations into psilocybin-assisted psychotherapy (PAP) for a wide variety of psychiatric disorders^5^. Several of the first modern trials of PAP focused on distress (such as anxiodepressive symptoms and existential suffering) associated with life-threatening illness^6–8^, given that its current treatments are limited^9^. That is, antidepressant medications demonstrate only small to moderate effect sizes in palliative care populations^10^. Psychotherapeutic interventions, including Cognitive-Behavioral Therapy as well as specialized existential-focused interventions^11^ such as dignity therapy^12^, may be effective in at least some symptom domains but have been subject to relatively little empirical study. Additionally, existing pharmacological and psychotherapeutic approaches generally require weeks or months to yield significant benefits, an important limitation in the setting of potentially rapidly-progressing diseases. In contrast, recent clinical trials of PAP have found safe, significant, and rapid improvements in both depression^13^ and anxiety^14^ for individuals facing life-threatening illnesses.

Much as the initial clinical studies of PAP have generated scientific excitement, they have also contributed to a global trend of reconsidering legal classifications of psilocybin to allow for varying degrees of legal access beyond clinical trials. Jurisdictions around the world have begun granting approval for the use of psychedelic substances for therapeutic purposes, though only Canada (since 2020) and Australia (through a ‘special access scheme’ in 2020, and through licensed healthcare providers since 2023) allow for compassionate access to psilocybin^15,16^. While the United States has not generally allowed for legal use of psilocybin, certain states and cities have decriminalized it, or at least deprioritized criminal enforcement for personal users^15^. Some have further moved towards legalization by authorizing psilocybin use in licensed centers under the supervision of licensed facilitators^17^.

The application of PAP clinical trial findings to clinical care in non-research contexts has resulted in a complex and rapidly-evolving landscape. As per the details on Canadian compassionate access pathways provided below, treatments outside of clinical trials are inevitably conducted in relatively heterogenous and uncontrolled conditions. For instance, treatment protocols, patient characteristics, and the nature or degree of psychological support, may all differ significantly in ‘real-world’ settings compared to the stringent trial protocols. Whether or not such differences may affect treatment safety or effectiveness is unknown.

### Details on the Canadian context

In Canada two pathways for legal access to psilocybin have been developed. The first involved the federal regulatory agency, Health Canada, beginning to grant personal exemptions to 'Subsection 56(1) of the Controlled Drugs and Substances Act in 2020. Such exemptions, often described as 'Section 56 exemptions', have been utilized for various medical, scientific, or public interest reasons and, in this case, allowed individuals to legally possess and consume psilocybin mushrooms. The second program, which has largely supplanted the Section 56 exemption pathway, occurs via the federal Special Access Program (SAP), which Health Canada amended to allow for licensed physicians and nurse practitioners to prescribe psilocybin. Both pathways permitted access to psilocybin for individuals suffering from serious or life-threatening conditions, including distress associated with terminal cancer diagnoses^18^.

The first Canadians to access psilocybin legally for therapeutic ends were four individuals with incurable cancer that were granted personal Section 56 exemptions by the federal health minister in August 2020^19^ Dozens of similar approvals followed in the ensuing years for Canadians with similar health conditions and, somewhat remarkably, for at least 17 healthcare professionals seeking firsthand experience with psilocybin in order to better provide care^20^.

Although groundbreaking, the Section 56 exemption pathway had numerous important issues. For one, it served only to decriminalize psilocybin for specific patients, but did not provide any means by which they could safely procure it. Additionally, these exemptions neither obliged nor facilitated any medical or psychological care accompanying the psilocybin. Indeed, healthcare professionals who facilitated PAP sessions for Section 56 exemptions faced significant medicolegal uncertainty regarding their involvement.

Following an initial wave of Section 56 approvals, these issues as well as lengthy delays in the evaluation of applications, prompted the Canadian federal government to amend the existing SAP to allow for another access pathway to psilocybin for therapeutic means. The SAP is a program in Health Canada which allows for authorized healthcare professionals (medical doctors or nurse practitioners) to request access to prescribe and administer certain drugs that are not yet approved for sale in Canada when more conventional treatment options have failed, are unsuitable, or are unavailable^21^. This program was commonly utilized, for example, to allow physicians to prescribe human immunodeficiency virus (HIV) treatments that had received regulatory approval in the United States but not in Canada^22^.

In January 2022, Health Canada amended the SAP to include psilocybin (as well as 3,4-Methylenedioxymethamphetamine [MDMA]) and granted multiple companies manufacturing and distribution licenses for standardized doses of psilocybin, either synthesized or as extracts. In contrast to Section 56 exemptions, applications to the SAP are completed by healthcare professionals requesting a specific drug, in a specific dose, obtained from an approved distributor and administered under their supervision. This has become the primary pathway utilized by healthcare providers to administer psilocybin to Canadian patients.

To our knowledge, licensed suppliers have generally provided psilocybin for use in the SAP without charge. In at least some cases, physicians have successfully billed the publicly-funded provincial insurance programs that provide universal healthcare in Canada^23^ for the time spent to provide therapeutic services, and potentially the associated administrative tasks. In other cases, clinicians have provided care ‘pro bono’, and/or charged fees directly to the patient or to their private insurance plans.

Although the SAP requires clear dosing protocols with medicolegal responsibility for safety assumed by psilocybin prescribers–neither of which were true of the Section 56 pathway–most nonpharmacological aspects of the PAP protocols are unspecified in both pathways. For instance, there are no requirements for the amount or type of psychological preparation, for the physical setting of the dosing session, or for the number and type of clinicians present.

To our knowledge, no data has been published on compassionate access programs despite their growing prevalence. This project was thus undertaken to address this gap. The study invited Canadians receiving legal access to PAP to voluntarily complete longitudinal surveys, before and after their psilocybin experience, to capture preliminary data on their characteristics and experiences in anticipation of expanding legal access to psilocybin and PAP.

## Methods

### Study design

We conducted a prospective longitudinal online survey inviting participation among Canadians who had received Section 56 exemptions to posses and consume psilocybin-containing mushrooms for the expressed intent of alleviating distress associated with life-threatening illness. The final sample of the study was restricted to participants who fully completed the baseline and endpoint surveys, as described below.

This work was performed in accordance with the Declaration of Helsinki and its later amendments, and was approved by Imperial College London’s Research Ethics Committee. Participants voluntarily registered to receive a series of electronic surveys via Imperial College London’s psychedelic survey platform (https://www.psychedelicsurvey.com/). The study was advertised by word of mouth and by the advocacy organization TheraPsil, which facilitated the legal applications of nearly all of the participants, by email and on their website.

### Recruitment

**Figure 1:**
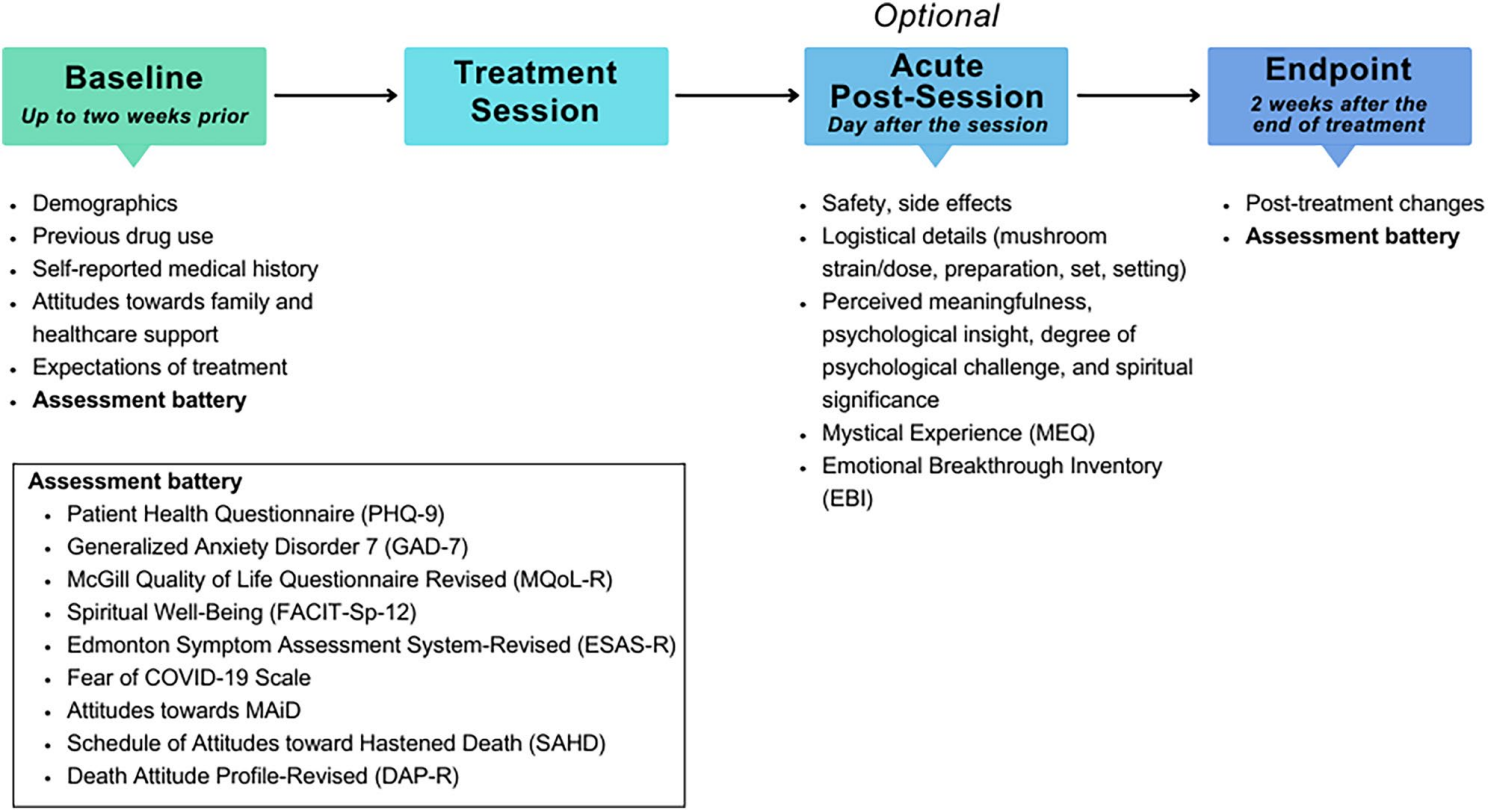
Timeline of survey completion.

Potential participants who came upon the study webpage were provided with information regarding the study. They were required to provide electronic consent and register with an email address, but otherwise no directly identifying information was collected. Registrants then received an email link to the baseline survey of the study, with additional surveys automatically sent to the same email addresses based on their provided treatment date (Fig. 1). All the participants provided an electronic informed consent for study participation. Participants were not compensated nor under any obligation to complete the surveys.

Recruitment occurred between December 2021 and July 2022. Fifty-one individuals registered for the study, provided electronic consent to participate, and completed at least a portion of the baseline survey. Of these, 16 participants completed the full baseline survey. Ten of these participants also completed the endpoint survey, two of whom were excluded because their responses indicated that they had not received Section 56 exemptions or received psilocybin. Thus, the final sample was comprised of eight participants who completed both the baseline and endpoint surveys in full, and whose detailed responses regarding their authorized legal access (as detailed in the baseline section below) were consistent with known facts of the compassionate access program, thus ensuring an administration of psilocybin in a formal therapeutic context. The Section 56 exemptions of the final study sample were granted between 11/05/2020 and 01/03/2022 (2020 n = 1; 2021 n = 6; 2022 n = 1).

## Measures

### Baseline

We collected data on participant demographics, self-reported, current and past psychiatric history and medical status, as well as details regarding substance use. The participants also provided information regarding their prior use of psychedelic substances (substance type, frequency of use, and within a ceremonial/therapeutic context), not including microdosing.

Details regarding their Section 56 exemption were requested, including the date of approval, the application process, any related costs or expenses, and the relevant medical condition. Additionally, the participants were asked to evaluate their family members/friends’ and healthcare team’s supportiveness regarding their upcoming psilocybin treatment (5-point Likert scale ranging from ‘Strongly disagree’ to ‘Strongly agree’). Finally, they were asked to evaluate their personal expectations of the treatment (5-point Likert scale ranging from ‘Major worsening’ to ‘Major improvement’) regarding symptoms of depression, physical pain, fear of death, spiritual well-being, relationships with family/friends, past trauma, or any other pertinent expectation (optional ‘other’ open text field).

### Baseline and endpoints assessment battery

A series of measures pertaining to depression and anxiety, quality of life, spiritual well-being, illness symptoms, attitudes towards MAiD and death, as well as fear of COVID-19 were administered both during the baseline as well as the endpoint survey.

#### Depression symptoms

Depressive symptoms were assessed using the Patient Health Questionnaire (PHQ-9^24^), a widely used 9-item self-evaluation of depressive symptoms assessed in the past 2 weeks (using a four-point Likert scale ranging from ‘Not at all’ to ‘Most days’).

#### Anxiety symptoms

Anxiety symptoms were assessed using the Generalized Anxiety Disorder-7 (GAD-7^25^), a 7-item self-evaluation of generalized anxiety disorder symptoms, which employs a similar time frame and Likert scoring as the PHQ-9.

#### Quality of life

The McGill Quality of Life Questionnaire Revised (MQoL-R^26^) is a revised version of the widely used assessment. It consists of 15-items which assess four domains (physical, psychological, existential, social) as well as an individual overall QoL item over the past 2 days. All items are scored using a 0–10 response scale (‘0’ being the worst and ‘10’ being the best).

#### Spiritual well-being

Spiritual well-being was assessed using the Functional Assessment of Chronic Illness Therapy—Spiritual Well-Being 12 Item Scale (FACIT-Sp-12^27^). The FACIT-Sp-12 probes aspects of spirituality and/or religious beliefs that the patient believes are beneficial to their health related quality of life over the past 7 days (using a five-point Likert scale ranging from ‘Not at all’ [0] to ‘Very much’[4]), yielding a total score as well as three subscale scores (Meaning, Peace, Faith^28^).

#### Illness symptoms

The patient’s perspective on their current illness symptoms were measured using the Edmonton Symptom Assessment System-Revised (ESAS-R^29^). Nine common symptoms are rated on a scale from 0–10 (‘0’ being the absence of the symptom and ‘10’ being the worst possible severity), including pain, tiredness, drowsiness, nausea, lack of appetite, shortness of breath, depression, anxiety, and well-being.

#### Attitudes towards MAiD

A self-constructed assessment of the patient’s attitudes towards medical assistance in dying (MAiD) were included in the survey, as MAiD is an accessible and growing option for Canadians facing life-threatening illnesses^30^. It consisted of three items (Likert scale ranging from ‘Strongly disagree’ [0] to Strongly agree [4]) pertaining to the likelihood (“I will likely receive Medical Assistance in Dying [MAiD] before my natural death.”), their certainty (“My mind is made up about my decision regarding Medical Assistance in Dying [MAiD]”, and their degree of peace (“I am at peace with my decision regarding Medical Assistance in Dying [MAiD]”) of receiving MAiD.

#### Attitudes towards death

General attitude towards death was assessed using the Death Attitude Profile-Revised (DAP-R^31^), which consists of a 32-item Likert scale (‘Strongly disagree’ [1] to [7]) with 5 subscales (Fear of Death, Death Avoidance, Neutral Acceptance, Approach Acceptance, Escape Acceptance). An additional measure was included to assess the participants’ desire for death (Schedule of Attitudes toward Hastened Death [SAHD]^32^). The SAHD consists of 20 true or false statements evaluating anticipated physical and emotional distress, as well as direct thoughts regarding the facilitation of one’s own death.

#### Fear of COVID-19

As this survey was conducted throughout the COVID-19 pandemic and surveyed vulnerable individuals, the Fear of COVID-19 Scale (FCV-19S^33^), a 7-item scale (5-point Likert scale ranging from ‘Strongly disagree’ [1] to ‘Strongly agree’ [5]), was used to assess the participants’ fear of COVID-19.

### Optional acute post-session timepoint

Participants were also sent an additional survey one day after their session. The survey included questions regarding the session details (logistics, safety, and treatment-related side effects), and questionnaires evaluating acute post-treatment changes. Session details included the form (and strain if known) of psilocybin consumed, dose, method of administration, session location, therapist credentials and past experience with psychedelics, pre-treatment preparation sessions (number and length), number of other people present during the session (therapists, other patients/participants), psychological or physical safety concerns, and the presence of various components. Data relating to the costs associated with the preparation and PAP sessions were also collected.

#### Revised mystical experience questionnaire 30 (MEQ30)

The MEQ30^34^ is a self-reported evaluation of psychedelic-induced mystical experiences. The 30 items are evaluated using a 6-point Likert scale (0 = ’None’ [not at all] to 5 = ’Extreme’ [more than any other time in my life]) and yield four factors (Mystical, Positive Mood, Transcendence of Time and Space, and Ineffability) as well as a total score. A ‘complete mystical experience’ is considered when at least 60% of the total possible score is reported for each factor^35^.

#### Emotional breakthrough inventory (EBI)

The EBI^36^ is a 6-item assessment of ‘emotional breakthrough’, including an evaluation of emotional release, a sense of relief and closure, and resolution of personal conflicts and traumas, confronting ignored emotions, and exploring challenging memories. Each item is rated on a visual analogue scale (VAS) ranging from 0 (‘No, not more than usually’) to 100 (‘Yes, entirely or completely’).

### Endpoint

#### Post-treatment changes

Similar to the baseline evaluation of personal expectations of the treatment, the participants were asked to reflect on their post-treatment changes relating to symptoms of depression, physical pain, fear of death, spiritual well-being, relationships with family/friends, past trauma, or any other pertinent expectation (optional ‘other’ open text field) using the same 5-point Likert scale.

### Data analysis

Participant characteristics were examined using descriptive statistics (frequency [n] and percentage [%]). Paired sample *t*-tests were used to test for changes in the assessment measures between baseline and at the two-week post treatment endpoint. The baseline and endpoint scores were examined for outliers, and normality was verified using the Shapiro–Wilk test. Four variables were found to be not normally distributed (p < 0.05), and a Wilcoxon signed-rank test was performed (Table S1, Supplemental material). The percentage (%) of ‘complete mystical experiences’ was also calculated (% of participants with each MEQ30 factor ≥ 60%). Statistical analyses were performed using JASP (Version 0.17.3). Raincloud plots were generated for each measure (baseline and endpoint) to visualize individual data points, boxplots displaying the median, lower quartile (25th percentile), and upper quartile (75th percentile), and one-sided violin plots depicting the overall data distribution.

## Results

### Participant characteristics

The final sample included eight individuals who completed the baseline and endpoint measures. Of the eight individuals, all were White, half were female (all reported the same gender as sex assigned at birth), and the sample mean age was 52.3 years (SD = 10.7). All participants were Canadian, with the majority being English speakers (7/8) and living in British Columbia (7/8). Further, the participants were either married (5/8) or single (3/8), and completed an average of 13 (SD = 7.7) years of education. The majority reported having a Master’s degree (3/8) or having completed trade/technical school (3/8), and were not currently working (unable to work [3/8], retired [2/8]). The total combined household income was varied, and ranged from under $25,000 (2/8) to > $125,001 (1/8); participants rated their position on a ladder relative to other Canadians as an average of 6.1 (SD = 1.4) out of 10 (with 10 being the ‘top’ people with the most money, the highest level of education, and the most popular jobs). Participant baseline characteristics are detailed in Table 1

**Table 1:** Baseline participant characteristics (n = 8).

Sociodemographics		
Age (mean + SD)		52.3 (10.7)
Sex assigned at birth (n,%)	Female	4 (50)
Gender (n,%)	Same as sex assigned at birth	8 (100)
Nationality (n,%)	Canadian	8 (100)
Province (n,%)	British Columbia	7 (88)
	Quebec	1 (13)
Native language (n,%)	English	7 (88)
	French	1 (13)
Race (n, %)	White	8 (100)
Relationship (n,%)	Married	5 (63)
	Single (never married)	3 (38)
Highest educational level attained (n, %)	Master’s degree	3 (38)
	Trade/Technical school	3 (38)
	Doctorate or professional degree (e.g., MD, PhD, Law Degree, JD)	1 (13)
	Less than high school	1 (13)
Years of education completed (mean, SD)		13.0 (7.7)
Employment (n, %)	Unable to work	3 (38)
	Retired	2 (25)
	Self-employed	2 (25)
	Homemaker	1 (13)
Total combined household income (CAD) (n, %)	$125,001 or over	1 (13)
	$100,001–CA$125,000	2 (25)
	Under CA$25,000	2 (25)
	Prefer not to say	3 (38)
Average rating of position on a socioeconomic ladder relative to others (0 to 10)		6.1 (1.4)

### *Life-threatening condition and section 56 exemption requests*

All participants reported having a life-threatening condition that was either palliative (4/8) or in remission (4/8). Among the four participants in remission, three 3/4 had been so for more than 3 months. All reported having a cancer diagnosis (unspecified [5/8], metastatic [1/8%], breast [1/8], small lymphatic lymphoma [1/8]). Two reported knowing their prognosis (2–3 years and 3–4 years).

The majority applied for the Section 56 exemption through a non-profit organisation or advocacy group (6/8), while the remainder filed the application independently or with help of a friend or family member (1/8) or applied through their doctor (1/8). Services related to the application process were reported to be paid for through the provincial health plan (3/8), privately by the applicant (3/8), or was provided pro bono by the therapist (2/8). The total estimated cost to the participant for the application process (CAD) ranged from $300 to $420 for those who reported paying out of pocket. Information regarding the participants’ life-threatening condition and Section 56 exemption requests are detailed in Table 2.

**Table 2:** Life-threatening condition, Section 56 exemption request, and treatment costs (n = 8).

Life-threatening illness information (n = 8)		
Condition (n,%)	Cancer, unspecified	5 (63)
	Cancer, metastatic	1 (13)
	Cancer, breast	1 (13)
	Cancer, Small lymphatic lymphoma	1 (13)
Condition was/is life-threatening (n,%)		8 (100)
Condition considered palliative or in remission (n,%)	Palliative	4 (50)
	Remission	4 (50)
In remission for more than 3 months (n = 4) (n,%)	No	1 (25)
	Yes	3 (75)
Prognosis, if known (n = 2) (n,%)	2–3 years	1 (50)
	3–4 years	1 (50)
Section 56 exemption application process and costs (n = 8)		
How did you apply for the Section 56 exemption? (n,%)	I filed the application independently or with help of a friend or family member	1 (13)
	Doctor	1 (13)
	Through a non-profit organisation or advocacy group (e.g., TheraPsil)	6 (75)
How were services related to the application process paid for (n,%)	Provincial health plan (most common method for physicians—no cost to me)	3 (38)
	Paid privately by me “out of pocket”	3 (38)
	Paid by my private insurance	0 (0)
	Partially or fully subsidized through compassionate pricing	0 (0)
	Therapists didn’t charge any fee to anyone that I’m aware of (it was “pro bono”)	2 (25)
How much was the total estimated cost to the participant (out of pocket) for the application process (CAD) (n, %)	$300	1 (13)
	$400	1 (13)
	$420	1 (13)
Costs related to the preparation and psychedelic-assisted psychotherapy sessions (n = 5*)		
How were services related to the preparation therapy sessions paid for (n,%)	Provincial health plan (most common method for physicians—no cost to me)	1 (17)
	Paid privately by me “out of pocket”	0 (0)
	Paid by my private insurance	0 (0)
	Partially or fully subsidized through compassionate pricing	3 (50)
	Therapists didn’t charge any fee to anyone that I’m aware of (it was “pro bono”)	2 (33)
How were services related to the psychedelic-assisted psychotherapy session paid for (n,%)	Provincial health plan (most common method for physicians—no cost to me)	1 (17)
	Paid privately by me “out of pocket”	0 (0)
	Paid by my private insurance	0 (0)
	Partially or fully subsidized through compassionate pricing	1 (17)
	Therapists didn’t charge any fee to anyone that I’m aware of (it was “pro bono”)	4 (66)

*One participant listed two sources covering preparation and treatment costs.

### Support and expectations

The participants described experiencing mostly positive support from family members/friends (strongly agree [4/8]; somewhat agree [2/8]; neither agree nor disagree [1/8]; strongly disagree [1/8])) as well as from their healthcare team (strongly agree [3/8]; somewhat agree [3/8]; strongly disagree [2/8]); though a minority reported strong disagreement. Most expected to experience a certain level of improvements in their symptoms of depression, fear of death, spiritual well-being, relationships, and past trauma, as well as minor improvement or no effects on their physical pain (Fig. 4).

### Current and past substance use, psychiatric conditions, and medication status

The participants provided detailed information regarding their current and past (month, lifetime) substance use (Table S2, Supplemental material). Most (7/8) were non-smokers (cigarettes), while half were current consumers of alcohol and cannabis. The participants reported a variety of past recreational drug use, with most reporting having used MDMA/ecstasy (5/8), cocaine (5/8), other (opiates, 1/8; ketamine, 1/8, GHB, 1/8), or none (1/8). Most reporting not using these substances within the past month.

The majority had previously consumed a psychedelic substance; the substances reported were varied, though the most common were psilocybin (6/8), LSD (5/8), and mescaline (3/8). The reported lifetime frequency of consumption of classic psychedelic substances (LSD, psilocybin, DMT, ayahuasca, mescaline), not including microdosing, was also varied, ranging from 10 times or less (2–5 times, 25%; 6–10 times, 2/8) to 11–20 times (2/8). Two participants reported prior use as high as 21–50 times and more than 100 times in their lifetime. Most had never taken a classic psychedelic substance in a therapeutic or ceremonial context (5/8), while three had minimal (2–5 times) to more extensive prior experiences (6–10 times, 11–20 times). Half (4/8) of the participants had not used psychedelic substances within the past 6 months (not including microdosing) or had used once (3/8).

The participants were queried about their current and past psychiatric history and diagnoses (Table S3, Supplemental material). The conditions reported included anxiety disorders (3/8), major depressive disorder (1/8), post-traumatic stress disorder (1/8), and anticipatory grief (1/8). None of the participants reported current or past diagnoses of a substance use disorder, eating disorder, bipolar disorder, attention deficit hyperactivity disorder (ADHD), autism spectrum disorder (ASD), personality disorder, psychotic disorder, hallucinogen persisting perception disorder (HPPD), or phobias (e.g. social anxiety disorder). Half (4/8) reported no psychiatric history or diagnoses.

### Change in measures between baseline and endpoint

**Figure 2:**
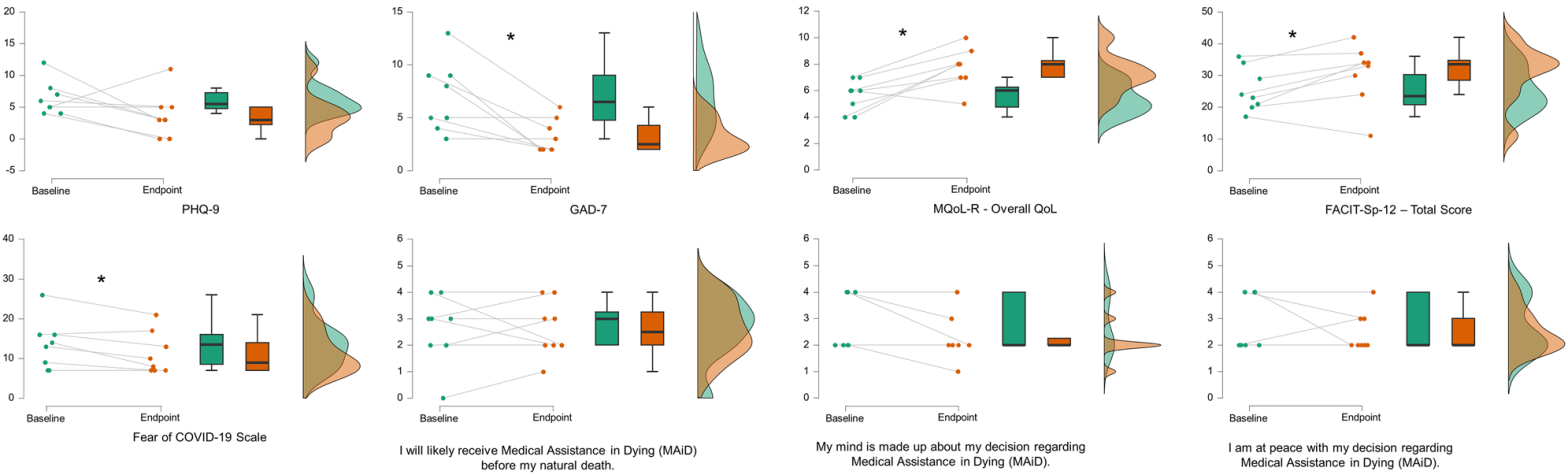
Raincloud plots displaying pre-post changes in the Patient Health Questionnaire-9 (PHQ-9), the Generalized Anxiety Disorder-7 (GAD-7), the McGill Quality of Life Questionnaire Revised (MQoL-R) Overall Score, the Functional Assessment of Chronic Illness Therapy—Spiritual Well-Being 12 Item Scale (FACIT-Sp-12) Total Score, the Fear of COVID19 scale, and the Medical Assistance in Dying (MAiD) questions. The dots depict individual data points, while the boxplots display the median, lower (25th percentile) and upper (75th percentile) quartile, and the one-sided violin plots display the overall distribution for both the baseline (green) and the endpoint (orange). * = p < 0.05.

Paired t-test results (mean ± standard error [SE], *t*, *p*, effect size [Cohen’s d]) for the changes in mood, quality of life, spiritual wellbeing, symptoms, fear of COVID, attitudes towards death, hastened death, and MAiD between the baseline and endpoint evaluations are detailed in Table 3**.** Significant decreased were observed for symptoms of anxiety (GAD7, ESAS-R), and depression (ESAS-R)pain, Fear of COVID-19, while significant increases were observed for overall QoL and psychological QoL (McGill QoL-R) as well as overall and peace measures of spiritual well-being (FACIT Spiritual Wellbeing). Figure 2 displays raincloud plots of pre-post changes in PHQ9, GAD7, MQoL, FACIT-Sp-12, Fear of COVID-19, and the self-constructed MAiD questions. Raincloud plots of pre-post changes in DAP-R and SAHD (Figure S1) and the ESAS-R (Figure S2) are displayed in the Supplemental Material.

**Table 3:** Change in measures between baseline and endpoint (n = 8).

Questionnaire		Baseline M (SE)	EndpointM (SE)	MD (SE)	*t*	*p*	ES
Mood	PHQ9	6.4 (0.9)	3.8 (1.3)	− 2.6 (1.6)	− 1.7	0.1	
	GAD7	7.0 (1.2)	3.3 (0.6)	− 3.8 (1.0)	− 3.6	0.008*	− 1.29
McGill QoL revised	Overall QoL	5.6 (0.4)	7.8 (0.5)	2.1 (0.6)	3.7	0.008*	1.29
	Physical QoL	6.6 (0.6)	7.6 (0.9)	1.0 (0.6)	1.8	0.1	
	Psychological QoL	5.3 (0.7)	7.0 (0.6)	1.6 (0.4)	4.7	0.002*	1.64
	Existential QoL	6.0 (0.4)	6.6 (0.4)	0.6 (0.4)	1.5	0.2	
	Social QoL	5.0 (0.2)	5.5 (0.4)	0.5 (0.5)	0.9	0.4	
FACIT spiritual wellbeing (FACIT-Sp-12)	FACIT total	25.5 (2.4)	30.6 (3.3)	5.1 (2.0)	2.5	0.04*	0.89
	FACIT meaning	11.1 (0.9)	12.1 (1.2)	1.0 (0.6)	1.6	0.2	
	FACIT peace	7.9 (0.7)	10.1 (1.0)	2.3 (0.8)	2.9	0.02*	1.03
	FACIT faith	6.5 (1.6)	8.4 (1.7)	1.9 (0.8)	2.3	0.06	
Edmonton symptom inventory (ESAS-R)	Pain	3.4 (0.7)	2.5 (0.8)	− 0.9 (0.2)	− 3.9	0.006*	− 1.37
	Tiredness	5.0 (0.7)	3.4 (0.8)	− 1.6 (0.8)	− 2.1	0.08	
	Drowsiness	3.1 (0.5)	3.1 (0.7)	0.0 (0.1)	0.0	1.0	
	Nausea	1.6 (0.6)	1.3 (0.2)	− 0.4 (0.5)	− 0.7	0.5	
	Appetite	2.3 (0.6)	1.6 (0.5)	− 0.6 (0.4)	− 1.7	0.1	
	SOB	3.0 (0.8)	1.8 (0.4)	− 1.3 (0.6)	− 2.2	0.06	
	Depression	5.0 (0.5)	2.9 (0.6)	− 2.1 (0.7)	− 2.9	0.02*	-1.01
	Anxiety	5.0 (0.6)	2.9 (0.4)	− 2.1 (0.7)	− 3.2	0.02*	-1.13
	Well-being	4.6 (0.3)	3.6 (0.6)	− 1.0 (0.6)	− 1.7	0.1	
Fear of COVID-19		13.5 (2.2)	11.3 (1.9)	− 2.3 (0.9)	− 2.6	0.04*	-0.90
Self-constructed MAiD questions	MAiD likelihood	2.6 (0.5)	2.6 (0.4)	0.0 (0.4)	0.0	1.0	
	MAiD certainty	2.8 (0.4)	2.3 (0.3)	− 0.5 (0.3)	− 1.9	0.1	
	Maid at peace	2.8 (0.4)	2.5 (0.3)	− 0.3 (0.3)	− 0.8	0.5	
Schedule of hastened death (SAHD)		5.5 (1.4)	5.4 (1.7)	− 0.1 (0.9)	− 0.1	0.9	
Death attitudes profile (DAP-R)	Fear of death	3.7 (0.1)	3.7 (0.2)	0.0 (0.2)	− 0.2	0.9	
	Death avoidance	3.6 (0.2)	3.6 (0.1)	− 0.1 (0.1)	− 0.5	0.6	
	Neutral acceptance	1.8 (0.2)	2.2 (0.3)	0.4 (0.2)	2.2	0.06	
	Approach acceptance	4.1 (0.1)	3.9 (0.2)	− 0.2 (0.3)	− 0.6	0.5	
	Escape acceptance	3.4 (0.2)	3.6 (0.3)	0.2 (0.3)	0.9	0.4	

*COVID-19* coronavirus disease 19, *FACIT* functional assessment of chronic illness therapy, *GAD* general anxiety disorder, *M* mean, *MAiD* medical assistance in dying, *MD* mean difference, *ES* effect size, *PHQ* patient health questionnaire, *QoL* quality of life, *SE* standard error of the mean, *SOB* shortness of breath.

* = p < 0.05.

### Acute experience and post-session evaluations

#### Session logistical details

Five participants completed the optional acute post session surveys evaluating safety, side effects, the session logistical details, the perceived meaningfulness, mystical experiences, and emotional breakthroughs.

All participants reported consuming the psilocybin in the form of psilocybin mushrooms or truffles (dried; 2/5 reported taking Golden Teacher [Psilocybe cubensis strain]; the other three reported not knowing). The majority reported taking only one dose ranging from 2.5 g (n = 1) to 5 g (n = 4) (one patient reported two doses over the course of the session as 3.33 and 1.66 doses, less than 1 h apart). Two participants consumed the psilocybin mushrooms/truffles whole, while the three others consumed it cooked or brewed into a tea. All reported having preparation/counselling sessions with their therapist(s) before the psilocybin administration (average of 3 sessions/5.8 h). The therapists involved in the psilocybin sessions included nine females (n = 5 registered nurses, n = 1 physician, n = 3 counsellors) and 5 males (n = 3 psychologist, n = 1 physician, n = 1 counsellor). The majority reported that the therapists had a past personal experience with psychedelics (Yes = 78.6%; Unsure = 21.4%), which the patients reported to have a mostly positive effect on their own experience (Very positive = 50.0%; Somewhat positive = 28.6%; No effect = 21.4%). Almost all had their session in a group setting, with more than 3 guides/facilitators/therapists providing supervision during their experience, except for one individual who had 2 present. For the preparation sessions, participants reported that the costs were either covered by the provincial health plan (1/5), partially or fully subsidized through compassionate pricing (1/5), or that the therapists didn’t charge any fee to anyone (3/5). For PAP sessions, the majority (4/5) reported that the therapists didn’t charge any fee to anyone, while one participant reported that their sessions were paid through the provincial health plan (Table 2).

#### Safety concerns, disruptions, therapeutic environment, presence of supports

Three participants described having no psychological or physical safety concerns at all during the session; one reported being somewhat concerned and another reported being extremely concerned. All reported an extremely conductive/supportive environments to their intentions. A variety of components were present during the sessions, including sacred/ritual objects (n = 3), personally meaning items/objects (n = 3), listening to recorded music (n = 5), the presence of emotionally supportive individuals (n = 4). One individual reported a disruption/distraction/interruption during their session, which was described as a perceived threat from others (imagined or ‘real’). None reported having family members or pets/animals present.

#### psilocybin-related side effects

The most common psilocybin-related side effects were nausea/vomiting (4/5), crying (3/5), headache (3/5), and sweat/chills (3/5) (Table 4).

**Table 4:** Side effects reported one day post-session (n = 5).

	n, %
Nausea or vomiting	4 (80)
Crying	3 (60)
Headache	3 (60)
Sweats or chills	3 (60)
Body aches	2 (40)
Trembling	2 (40)
Diarrhea	1 (20)
Ringing in the ears	1 (20)

### Personal meaningfulness, mystical experiences, and emotional breakthroughs

**Figure 3:**
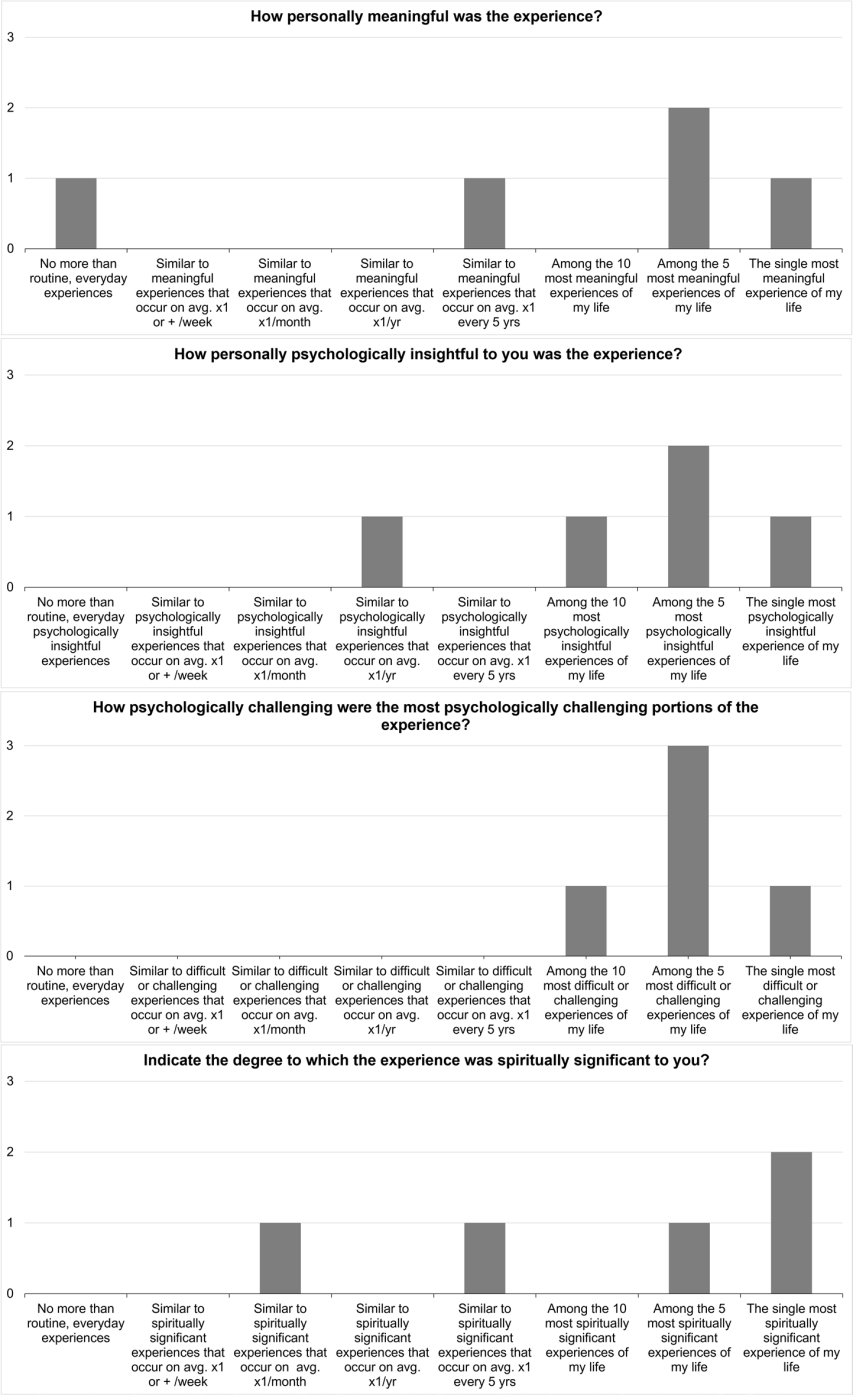
Post-treatment evaluation of the psychological insight, meaningfulness, spiritual significance, and degree of psychological challenge of the experience.

The post-treatment evaluation of personal meaningfulness of the experience varied, ranging from being described as the single most meaningful experience of their life (1/5) to being no more than routine, everyday experiences (1/5). Two participants found their experience to be the single most spiritually significant experience of their lives, and one found it to be the single most psychologically insightful experience of their life. All found some part of the session to be psychologically challenging, though this also ranged from being the single most difficult or challenging experience of their life (1/5) to among the top 5 (3/5) and top 10 (1/5) most difficult or challenging experiences of their lives. The evaluation of the experiences’ psychological insight, meaningfulness, spiritual significance, degree of psychological challenge are presented in Fig. 3.

The mean total score of the MEQ30 was 64.3 (± 23.6), with 2/5 participants having a ‘complete mystical experience’ (i.e., ≥ 60% on all MEQ30 factors). The mean total score of the EBI was 63.4 (± 27.7), with the highest variable being “*I faced emotionally difficult feelings that I usually push aside.”* (89.2 [± 14.7]). Total scores and subscales for the EBI and MEQ30 are reported in Table S4 (Supplemental material).

### Endpoint—evaluation of self-assessed treatment-related changes

**Figure 4:**
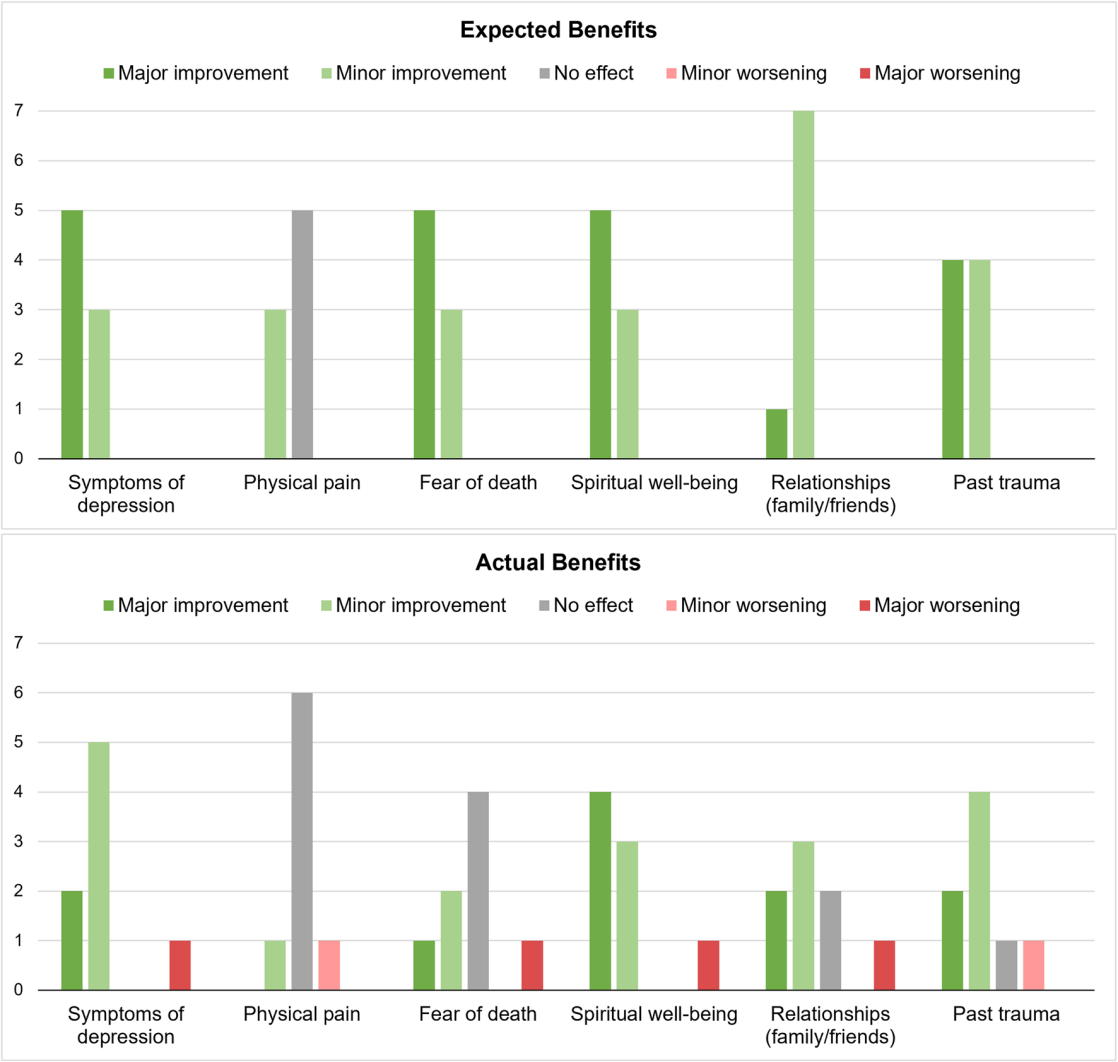
Comparison of pre-treatment expected benefits to actual, post-treatment benefits.

Two weeks post-treatment, the participants reflected on their perceived treatment-related changes in depression, physical pain, spiritual well-being, relationships, and past trauma. Most experienced a degree of improvement in their symptoms of depression, spiritual well-being, relationships, and past trauma with one notable exception describing a ‘major worsening’ of these aspects of their lives. Most (75%) did not experience any effect on their physical pain, while half experienced no effect on their fear of death (Fig. 4).

## Discussion

This prospective longitudinal study aimed to provide a preliminary evaluation of the experiences of individuals suffering from distress associated with life-threatening illness who received compassionate access to psilocybin with psychological support. Eight individuals with life-threatening cancer diagnoses, either palliative or in remission, completed the baseline and follow-up surveys two weeks after the administration of psilocybin mushrooms in a supportive naturalistic setting, demonstrating significant decreases in reported symptoms of anxiety and depression, pain, as well as increases in overall quality of life and spiritual well-being. These findings are largely in line with controlled clinical studies of psilocybin for end-of-life depression^13^ and anxiety^14^, including the alleviation of state anxiety and symptoms of depression in individuals with cancer diagnoses, and yielding longer-lasting (two weeks post-treatment) benefits. No studies reported any serious adverse effects following psilocybin administration. It is noteworthy, however, that serious safety concerns were reported by two participants in this current report, and one out of eight participants reported major worsening across several relevant psychological domains, indicating that the lack of standardisation and regulation of compassionate access to psychedelic services present in Canada at the stage of data collection might carry health risks exceeding those in controlled clinical psilocybin use. Risks may arise due to several factors, including the lack of clearly defined therapist training, the absence of standardized symptom and safety assessments, and less stringent inclusion criteria compared to clinical trials, where screening failure rates are high.

The results demonstrate ways in which real-world psilocybin use differs from comparable clinical trials. Consistent with the Section 56 exemption pathway, participants consumed psilocybin mushrooms, either raw or cooked/brewed, rather than synthetic psilocybin or standardized extracts as typically used in clinical trials. In the current study, the most prevalent side-effects associated with the psilocybin consumption included nausea/vomiting, crying, headaches, and sweat/chills, though participants also reported body aches and trembling as well as diarrhea and ringing in the ears. These generally mirror psilocybin-related adverse events reported in 27 clinical trials, with the most commonly reported symptoms being headaches, elevated blood pressure (not assessed in the current work as it was self-reported recall), nausea, and anxiety (not assessed as a side effect in our sample, though ‘crying’ was common)^37^. However, only 3/27 of these trials were conducted with individuals suffering from distress associated with life-threatening illnesses. Additional fairly common physical side effects reported in the current study included sweat or chills, body aches, and trembling; considering the small sample, additional work is needed to examine specific side effects reported in these more vulnerable populations who are suffering with somatic symptoms in addition to their psychological distress.

The findings align reasonably well with results from clinical trials examining psilocybin as a treatment for depression and anxiety, including distress associated with life-threatening illness. Significant improvements in symptoms of anxiety, assessed using both the GAD7 (> 50% reductions) and the ESAS-R, along with increases in overall quality of life and spiritual well-being were observed between baseline and the endpoint. Although there wasn’t a significant reduction in depression scores measured by the PHQ9 (possibly due to a basement effect), significant decreases in depressive symptoms assessed through the ESAS-R were observed. Two recent meta-analyses and systematic reviews found that psilocybin was superior to placebo in alleviating state and trait anxiety, as well as symptoms of depression in this population, yielding both acute and longer-lasting benefits^13,14^. Sustained improvements in quality of life, life meaning, death acceptance, and optimism were also found in the largest RCT of psilocybin for distress associated with life-threatening illness to-date (n = 51)^6^. Additionally, while this is the first report of PAP decreasing fear of COVID-19 in individuals facing life-threatening illnesses, this finding may be explained through an overall decrease in anxiety symptoms observed in these individuals, or through global decreases in fear of COVID-19 over time.

Perhaps surprisingly, there were no observed changes in the participants’ attitudes towards death or MAiD, or towards a desire for hastened death. To our knowledge, this is the first examination of the intersection between MAiD and PAP in individuals facing life-threatening illnesses. The results indicated that the participants did not change their minds about their likelihood of receiving MAiD, their certainty, and their degree of peace towards that decision, despite experiencing decreases in symptoms of depression, anxiety, physical pain, and increases in spiritual well-being and quality of life. This result runs contrary to previous research findings of greater degree spiritual well-being being associated with a decreased desire for hastened death^38^. Consequently, the enhancements in spiritual well-being noted in the present study may not translate into reduced desires for hastened death, at least within the study’s relatively brief timespan.

While the overall changes observed between baseline and the two-week endpoint were positive, the acute post-session data indicated that there was a variety of experiences relating to the meaningfulness and importance of the experiences, as well as the reported mystical experiences and emotional breakthroughs that should be examined. The mean total MEQ30 score was 64.3, which matches previous reports with high dose synthetic psilocybin (22 mg or 30 mg/70 kg) in a comparable sample of cancer patients with life-threatening diagnoses and symptoms of depression and/or anxiety^6^. The mean EBI score was 63.4; slightly higher than what was observed in the initial validation study (M = 43 ± 31.5^28^). Notably, the majority of participants felt that their treatment helped them to face emotionally difficult feelings that they usually push aside (M = 89.2 ± 14.7) and that they felt able to explore challenging emotions and memories (69.4 ± 33.3). However, this has never been assessed in a sample of individuals suffering from distress associated with life-threatening illness, and more research is required.

Some participants recalled their session as being the single most meaningful (n = 1), spiritual (n = 2), or psychologically insightful (n = 1) experience of their lives. All the participants found some part of the session to be psychologically challenging, and indeed one individual described it as being the single most difficult/challenging experience of their life. While these findings are preliminary and drawn from a limited sample, they highlight the fact that difficult and challenging experiences are possible, and that careful preparation, monitoring, and integration are required when conducting PAP sessions.

Overall, expectations of treatment benefits prior to the experience were high (i.e. most were expecting minor to major improvements), reflective perhaps of the promising findings from clinical trials and related media reports. Indeed, the risk of biases and the possible confounding effects of expectations are prominent concerns in this research field^39^. All participants expected the psilocybin session to yield major or minor improvements in depression, fear of death, spiritual well-being, relationships, and past trauma. After the psilocybin session, reported benefits were generally more modest and crucially, one participant reported major worsening in depression, fear of death, spiritual well-being, and social relationships. Together, these findings underline the importance of expectation management when applying novel interventions in real-world settings.

The limited ecological validity of small, thoroughly screened samples undergoing psilocybin treatments in highly controlled hospital conditions effectively means that little to nothing is currently known about the risks of widespread adoption of psilocybin services in heterogeneous populations and settings, as our results have demonstrated. Observational studies such as the one presented here will therefore be crucial in order to evaluate and improve the safety and harm-benefit profile of psychedelic interventions once they leave the safe perimeter of clinical research. Use of the compassionate access pathways for PAP would greatly benefit from adopting the same high standards of psychological support and risk assessments currently employed in clinical trials, given adequate training and resources. Additionally, patients accessing psilocybin through these pathways are largely required to pay out of pocket for their treatment if they do not receive subsidizations from their healthcare providers, therapists, or coverage from their provincial or private insurers. In the current sample, while some participants paid for their application costs, their preparation and psilocybin treatment sessions were primarily covered ‘pro-bono’. These findings highlight the crucial role played by dedicated healthcare workers and therapists who have volunteered their time to make this treatment available to their suffering patients. However, on a larger scale, this approach is not sustainable. If approved, the scalability of this treatment will need to be carefully considered.

## Limitations

The most notable limitation of the current findings is the modest and homogenous sample size. Despite several dozen Canadians receiving access to PAP during the data collection phase of this survey, recruitment was a major challenge. This is likely a consequence of limited advertising of the project—a choice intended to decrease the chances of ineligible participants signing up—as well as the lack of in-person contact and the fully optional nature of the project. Additional challenges which may have contributed to our limited sample size include the disconnect of care providers across a large country with different provincial health systems, a lack of incentives for participations beyond altruistic support for the movement, the health status of potential participants (physical and psychological challenges), a possible lack of technological access/abilities to complete digital surveys, a hesitancy to share email addresses/contact information given the illicit status of psilocybin, and the possibility that patients who have difficult experiences may be unwilling to share them with a research team.

This issue attests to the need for more formal programs to surveil and study the experiences of patients receiving compassionate access to PAP, which remains an experimental treatment despite the worldwide loosening of regulations surrounding psychedelic medicines. The lack of obligatory or voluntary data collection pathways for patients receiving PAP through compassionate access may represent a missed opportunity to further understand this novel intervention in advance of expected regulatory approvals in the coming years. Suggestions for better data collection and reporting on these individuals may include a central registry of patients who have been granted Section 56 exemptions/SAP approvals for psilocybin with mandatory minimum (anonymized) data elements and the creation of a network of healthcare providers and therapists who are involved in these treatments for improved research integration and treatment standardization.

Additional limitations of these findings are the anonymity of the survey participants (though the data was screened and responses were cross-referenced to verify the veracity and plausibility of the responses), the open-label nature of the PAP sessions, the lack of standardization of all aspects of the individualized PAP treatment received, and the lack of data on previous ceremonial use or microdosing use. Additionally, future work would benefit from more detailed data collection regarding the conduction of the psilocybin treatment sessions.

## Conclusion

We present the first preliminary data, to our knowledge, examining the experiences of patients receiving compassionate access to psilocybin—in this case, for Canadians with distress associated with life-threatening illness. This initial investigation provides detailed descriptions of changes in mental health symptoms, treatment expectations, and safety information derived from the experience of a single ‘real-world’ PAP session. Therapists who are supporting individuals considering undergoing this treatment in the near future, whether via compassionate access programs or clinical trials, can utilize these findings by gaining insight into the potential benefits and challenges, and by facilitating discussions on treatment expectations and the potential for encountering difficulties during the process.

Despite the increasing interest and accessibility of psilocybin for a variety of psychiatric conditions, there have been no formal evaluations of the experiences of individuals receiving government sanctioned, compassionate access to psilocybin. Our results, including the challenges of recruitment, underscore the need for establishing formal surveillance programs in conjunction with compassionate access programs, not only within Canada but also globally as more countries allow the therapeutic use of psychedelic medicines.

### Supplementary Information

Supplementary Information.

## Data Availability

The data that support the findings of this study are not openly available due to patient privacy but are available from the corresponding author upon reasonable request.
