# Supplementary material for: Longitudinal experiences of Canadians receiving compassionate access to psilocybin-assisted psychotherapy
Source: Sci Rep. 2024 Jul 17;14:16524. doi: 10.1038/s41598-024-66817-0 (PMC11254907; doi:10.1038/s41598-024-66817-0)
Supplement: Supplementary file 1 — Supplementary Information. [file 41598_2024_66817_MOESM1_ESM.docx]

**Supplementary Information**

**Longitudinal experiences of Canadians receiving compassionate access to psilocybin-assisted psychotherapy**

**Supplementary Tables**

**Table S1.** Results from the Wilcoxon sign-ranked test for the four variables which were found to be non-normally distributed (Edmonton Symptom Inventory: Pain, Nausea, Appetite, Well-being; Death Attitudes Profile: Death Avoidance)

|  |  | **W** | ***z*** | ***p*** |
| --- | --- | --- | --- | --- |
| **Edmonton Symptom Invent.** | Pain | 0.0 | -2.2 | 0.03 |
|  | Nausea | 1.0 | -0.4 | 1.0 |
|  | Appetite | 0.0 | -1.6 | 0.2 |
|  | Well-being | 8.0 | -1.4 | 0.2 |
| **Death Attitudes Profile (DAP-R)** | Death Avoidance | 4.0 | -0.3 | 0.9 |

*p* significance value; *W* Wilcoxon sign-ranked test; *z* z score

**Table S2.** Participant substance use (n=8)

| Substance consumed | | |
| --- | --- | --- |
| Cigarette use (n,%) |  | 1 (13) |
| How many cigarettes smoked per day on average over the past month (Cigarette smoker only) | 1 | 1 (100) |
| Alcohol use (n,%) |  | 4 (50) |
| Last time drank alcohol (Alcohol drinkers only) (n, %) | Between 1 and 2 weeks ago | 1 (25) |
|  | Within the last week | 3 (75) |
| Alcohol consumed in a typical week over the past month (n,%) | 1 - 6 units per week (up to 2 pints of lager OR 2 large glasses of wine OR 2 double measures of spirit) | 3 (75) |
|  | 19 - 24 units per week (up to 8 pints of lager OR 8 large glasses of wine OR 8 double measures of spirit) | 1 (25) |
| Cannabis use (n,%) |  | 4 (50) |
| Over the past month, how often did you use cannabis on average? (n,%) | 4 or more times a week | 2 (50) |
|  | Monthly or less | 2 (50) |
|  | | |
| Psychedelic substances consumed in the past (n,%) | Psilocybin / magic mushrooms / truffles | 6 (75) |
|  | LSD | 5 (63) |
|  | Mescaline (Peyote, San Pedro) | 3 (38) |
|  | Ayahuasca | 2 (25) |
|  | Hallucinogen-type NPS (e.g. 25I-NBOMe/N-Bomb, 2C-B, 4-ACO-DMT, 1P-LSD) | 2 (25) |
|  | DMT | 1 (13) |
|  | Salvia Divinorum | 1 (13) |
|  | None of the above | 1 (13) |
|  | | |
| Number of times taken classic psychedelic substances in entire life (LSD, psilocybin, DMT, ayahuasca, mescaline), not including microdosing. (n,%) | 11-20 times | 2 (25) |
|  | 2-5 times | 2 (25) |
|  | 6-10 times | 2 (25) |
|  | 21-50 times | 1 (13) |
|  | More than 100 times | 1 (13) |
|  | | |
| Frequency of use of classic psychedelic substances in a therapeutic or ceremonial context (LSD, psilocybin, DMT, ayahuasca, mescaline), not including microdosing (n,%) | Never | 5 (63) |
|  | 11-20 times | 1 (13) |
|  | 2-5 times | 1 (13) |
|  | 6-10 times | 1 (13) |
| Frequency of use of psychedelic substances in the past month, not including microdosing (n,%) | Not within the past 6 months | 4 (50) |
|  | Once | 3 (38) |
|  | Missing | 1 (10) |
|  | | |
| Other reported substances consumed recreationally at least once (n,%) | MDMA/Ecstasy | 5 (63) |
|  | Cocaine | 5 (63) |
|  | Amphetamines | 2 (25) |
|  | Opiates | 1 (13) |
|  | Ketamine | 1 (13) |
|  | GHB | 1 (13) |
|  | None of the above | 1 (13) |
|  | Benzodiazepines | 0 (0) |
| Frequency of use over the past month (n,%) | | |
| Amphetamines (n=2) | Not at all within the past month | 2 (100) |
| MDMA/Ecstasy (n=5) | Not at all within the past month | 5 (100) |
| Cocaine (n=5) | 6-10 times | 1 (20) |
|  | Not at all within the past month | 4 (80) |
| Opiates (n=1) | Not at all within the past month | 1 (100) |
| Ketamine (n=1) | Once | 1 (100) |
| GHB (n=1) | Not at all within the past month | 1 (100) |

*DMT* N,N-Dimethyltryptamine; *GHB* gamma hydroxybutyrate; *LSD* lysergic acid diethylamide; *MDMA* 3,4-Methyl​enedioxy​methamphetamine; *NPS* new psychoactive substances

**Table S3.** Psychiatric history and medication status (n=8)

|  | | |
| --- | --- | --- |
| Reported Psychiatric Illnesses | Anxiety disorder or obsessive-compulsive disorder | 3 (38) |
|  | Major depressive disorder | 1 (13) |
|  | Post-traumatic stress disorder | 1 (13) |
|  | Other: Anticipatory grief | 1 (13) |
|  | None of the above | 4 (50) |
| Reported Medications (Past Year) | | |
| Antidepressants | Currently taking regularly | 0 (0) |
|  | Currently taking as needed | 0 (0) |
|  | Prescribed in past year but not currently taking | 3 (38) |
| Antipsychotics | Currently taking regularly | 0 (0) |
|  | Currently taking as needed | 0 (0) |
|  | Prescribed in past year but not currently taking | 1 (13) |
| Benzodiazepines | Currently taking regularly | 0 (0) |
|  | Currently taking as needed | 0 (0) |
|  | Prescribed in past year but not currently taking | 1 (13) |
| Cannabis | Currently taking regularly | 1 (13) |
|  | Are you planning to stop taking cannabis ahead of your psychedelic treatment? No =1 |  |
|  | Currently taking as needed | 0 (0) |
|  | Prescribed in past year but not currently taking | 1 (13) |
| Opioids | Currently taking regularly | 0 (0) |
|  | Currently taking as needed | 0 (0) |
|  | Prescribed in past year but not currently taking | 2 (25) |
| Stimulants | Currently taking regularly | 0 (0) |
|  | Currently taking as needed | 0 (0) |
|  | Prescribed in past year but not currently taking | 1 (13) |

**Figure S1.** Raincloud plots displaying pre-post changes in the Death Attitude Profile-Revised (DAP-R) and the subscales (Fear of Death, Death Avoidance, Neutral Acceptance, Escape Acceptance, Approach Acceptance), and the Schedule of Attitudes toward Hastened Death (SAHD). The dots depict individual data points, while the boxplots display the median, lower (25th percentile) and upper (75th percentile) quartile, and the one-sided violin plots display the overall distribution for both the baseline (green) and the endpoint (orange). *=p<0.05.

**
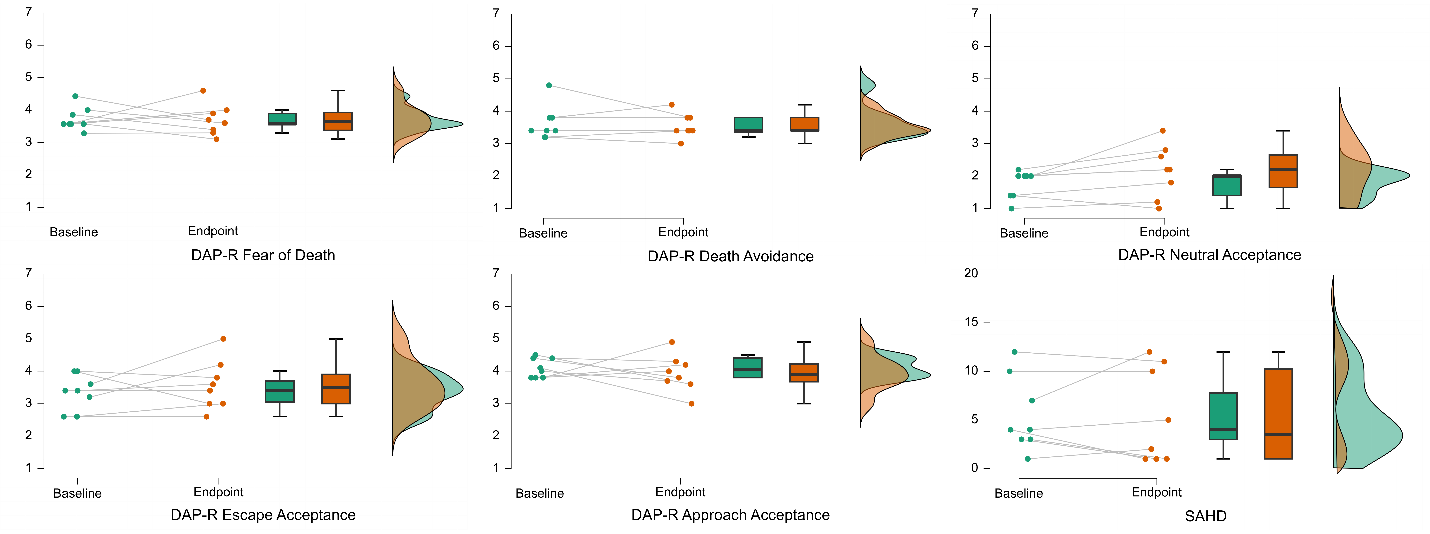
**

**Figure S2.** Raincloud plots displaying pre-post changes in the Edmonton Symptom Inventory (ESAS-R) and the subscales (Tiredness, shortness of breath [SOB], Depression, Well-being, Anxiety, Drowsiness, Pain, Lack of appetite, and Nausea). The dots depict individual data points, while the boxplots display the median, lower (25th percentile) and upper (75th percentile) quartile, and the one-sided violin plots display the overall distribution for both the baseline (green) and the endpoint (orange). *=p<0.05.

**
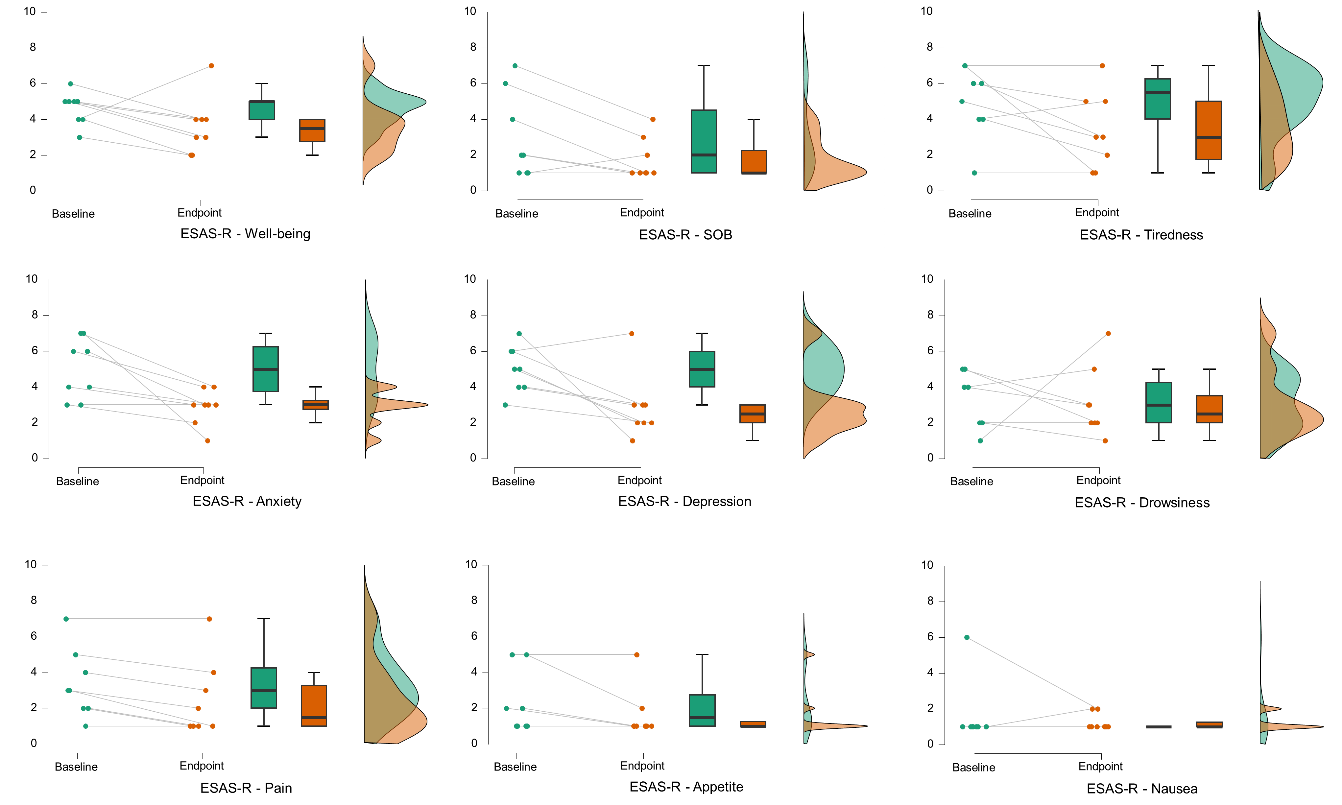
**

**Table S4.** Mystical experiences, and emotional breakthrough (n=5)

| **Mystical Experience Questionnaire (MEQ30)** **(M±SD)** |  |
| --- | --- |
| Total score | 64.3 (23.6) |
| Mystical subscale | 64.3 (22.3) |
| Positive mood subscale | 62.7 (24.5) |
| Transcendence subscale | 64.7 (27.6) |
| Ineffable subscale | 66.6 (26.2) |
|  |  |
| Complete mystical experience* (n, %) | 2 (40.0%) |
|  | |
| **Emotional Breakthrough Inventory (EBI) (M±SD)** |  |
| Total score | 63.4 (27.7) |
| I faced emotionally difficult feelings that I usually push aside. | 89.2 (14.7) |
| I experienced a resolution of a personal conflict/trauma | 53.4 (38.3) |
| I felt able to explore challenging emotions and memories. | 69.4 (33.3) |
| I had an emotional breakthrough. | 67.4 (31.0) |
| I was able to get a sense of closure on an emotional problem. | 48.6 (48.3) |
| I achieved an emotional release followed by a sense of relief. | 52.2 (47.3) |

** Indicates the percentage of participants whose scores on all MEQ30 subscales were at least 60% of the highest possible score.*
